# Functional assignment to hypothetical proteins in *Orientia tsutsugamushi*strain Ikeda

**DOI:** 10.6026/97320630018188

**Published:** 2022-03-31

**Authors:** Dixit Sharma, Sunil Kumar, Ankita Sharma, Rakesh Kumar, Ranjit Kumar, Mahesh Kulharia, Manish Kumar

**Affiliations:** 1 Department of Animal Sciences, School of Life Sciences, Central University of Himachal Pradesh, District Kangra, Himachal Pradesh, India - 176206; 2 Centre for Computational Biology and Bioinformatics, School of Life Sciences, Central University of Himachal Pradesh, District Kangra, Himachal Pradesh, India - 176206; 3 Department of Chemistry and Chemical Sciences, School of Physical and Material Science, Central University of Himachal Pradesh, District Kangra, Himachal Pradesh, India - 176206

**Keywords:** Orientia tsutsugamushi, hypothetical proteins, therapeutic targets

## Abstract

*Orientia tsutsugamushi*(*O. tsutsugamushi*) is an intracellular bacterial pathogen which causes zoonosis scrub typhus in humans. Genome of *O. tsutsugamushi* strain Ikeda contains 214 hypothetical proteins (HPs) which is nearly 20% of the total proteins. Domain and family based functional analysis of HPs results in the annotation of 44 hypothetical proteins. The annotated HPs were classified in to five main classes namely, gene expression and regulation, transport, metabolism, cell signaling and proteolysis. Thus, computational analysis of HPs helps to understand their putative roles in various biological and cellular processes, including pathogenesis for further consideration as potential therapeutic targets.

## Background:

*Orientia tsutsugamushi*(*O. tsutsugamushi*) is an obligate intracellular pathogen responsible for infectious illness scrub typhus in humans. The zoonotic disease scrub typhus is a vector borne disease transmitted to human through the bite of Leptotrombidium mite larvae. The disease is predominant in Asia-Pacific region but the cases from Middle East and South America showed the wide geographical distribution of pathogen [1-2]. Scrub typhus in South East Asia is most important emerging infectious zoonotic disease and most common rickettsial disease in India. The outbreak of the disease was reported in different provinces in India including Assam, Himachal Pradesh, Jammu and Kashmir, Bihar, Rajasthan, Tamilnadu and may more [3]. The infection of *O. tsutsugamushi* can also lead to multiorgan failure and other disease like neurological complications, Parkinson's disease, acute kidney injury and acute respiratory distress syndrome [4-6]. Due to high mortality and non-specific representation of disease the effectual control measure are needed. Earlier attempts were made to prioritize metabolic pathway proteins and metal-binding proteins of *O. tsutsugamushi* as potential therapeutic targets [7-8]. The experimental techniques are very useful but expensive and time consuming whereas computational techniques evolved as most promising and reliable approaches for dissecting the genome or proteome sequence datasets[9,10]. Earlier, computational methods were efficiently used for deciphering the whole proteome data of different bacterial pathogens [11,12]. Therefore, it is of interest to document data on the functional assignment to hypothetical proteins (HPs) in *Orientia tsutsugamushi*strain Ikeda for further consideration in drug development.

## Methodology:

### Hypothetical proteins dataset retrieval:

The complete proteome dataset of *O. tsutsugamushi* strain Ikeda was downloaded from Ref Seq server. Further, the HPs were extracted by searching the term "hypothetical proteins" in the whole proteins dataset.

### Functional prediction:

Functional conserved signature sequences or domains of HPs were predicted by Inter Pro Scan [13] and Pfam [14]. Inter Pro Scan allows protein sequence to search against Inter Pro's signatures i.e., predicted models that define families, domains and sites of the proteins. Pfam is the collection of curated protein families with clearly described organization of domains.

### Metabolic pathway analysis:

Blast KOALA [15] at Kyoto Encylcopedia of Genes and Genomes (KEGG) was used for the analysis of metabolic pathways of HPs. KEGG is integrated database for biological interpretation of genomic and other high throughput sequence data.

### Sub-cellular localization prediction:

The subcellular localization of HPs were investigated by SOSUIGramN [16]. SOSUIGramN predict subcellular localization on the basis of physiochemical parameters of N- and C-terminal signal sequence and total sequence. The overall accuracy of the software is 92.9%.

### Identification of potential therapeutic targets among HPs:

To extract non-homologous proteins of *O. tsutsugamushi*, BLASTp [17] search of HPs was performed against host (human; taxid: 9906) proteins at NCBI with expect value (E-value) 10-5. Bacterial toxins are described as most potent targets for drug discovery process. Essentiality of HPs was checked with the help of Database of Essential Genes (DEG) version 10 [18]. The DEG harbors essential genes and proteins, regulatory sequences, noncoding RNAs, promoters and replication origins. The BLASTp search of the HPs was performed against DEG database with cut-off parameter of 0.00001 E-value. The selected hypothetical proteins having homologous hits with the DEG proteins database were considered as essential. Expasy's Prot Param [19] was used for the physical and chemical parameters analysis of HPs from *O. tsutsugamushi*. The identification of physicochemical properties of proteins provides insight into their biochemical nature and serves as the initial step for the studies like drug discovery. Further, the prediction of bacterial toxins among HPs was performed by BTXpred [20]. BTXpred is the online server to predict bacterial toxins with overall accuracy of 96.07%.

## Results and Discussion:

The vector borne zoonotic disease scrub typhus caused by an intracellular pathogen *O. tsutsugamushi* is most remarkable neglected tropical disease having ever-widening impact, worldwide. Studies have even shown that in addition to scrub typhus *O. tsutsugamushi* can lead to various other complications [4,5]. Hypothetical proteins are the proteins with known amino acid sequences but with unknown functions. Earlier studies showed the involvement of HPs in different biological processes including drug development [5]. The whole proteome analysis of *O. tsutsugamushi* strain Ikeda showed that about 20% of the total proteins are hypothetical. We have functionally annotated and classified 44 HPs based on conserved functional domain and family. The 44 functionally annotated HPs were classified in to five broad classes, i.e., gene expression and regulation, cell signaling, metabolism, transport and proteolysis. The detailed description of the study is mentioned below.

The *O. tsutsugamushi* strain Ikeda proteome was publically available at RefSeq with genome reference number NC_010793.1. The size of the genome is 2.01 Mb with 30.5% GC content. The proteome of *O. tsutsugamushi* contains 1227 proteins out of which 241 were HPs (Figure 1 - see PDF). The 241 HPs were extracted from the whole proteome and selected for further analysis. The HPs are encoded by open reading frames but they do not have any known function. In many pathogenic bacteria, HPs play crucial roles in pathogens survival and advancement of related infectious diseases [23]. The hypothetical proteins were annotated on the basis of conserved domain and family. We have identified the function of 44 HPs and classified in to five broad classes, i.e., gene expression and regulation (25), transport (9), metabolism (5), cell signaling (4) and proteolysis (1) (Figure 2 - see PDF). The main domains present in the HPs were DnaA N-terminal domain, Outer membrane enzyme PagP, Integrase and Group II intron (Figure 3 - see PDF). Genome sequencing of *O. tsutsugamushi* showed that it contains immense number of repetitive sequences [24]. In our present study we find the existence of integrase, ankyrinrepeat and DnaB-like proteins which belong to the category of repetitive sequences. The in vivo gene expression of *O. tsutsugamushi* was studied earlier and identified genes found to involved in DNA replication, protein translation, post translational modification, signaling, transport and metabolism [25]. The study strengthens our results as we have also observed the presence of proteins in class of gene expression and regulation, cell signaling, metabolism and transport.

The category of gene expression and regulation showed the presence of DnaA N-terminal domain, integrase, group II intron, transposase DDE domain, DnaB-like, Leucine-rich repeat domain, Phage gp-6-like, ribosomal protein S2, RNA polymerase, ribonuclease reductase-like and ribonuclease H superfamily (Figure 3, Table 2 - see PDF). N-terminal domain is crucial for the activity of bacterial DnaA at the origin of replication. It is involved in the coordination of chromosomal replication with cell cycle, cellular processes and oxidative stress [26]. Replicative DnaB-like helicases are required for unwinding of DNA strand and important in the movement of replication fork direction. The C-terminal region of DnaB helicases is vital for proper folding and contains motifs necessary for helicases activity [27]. Furthermore integrase mediates specific cut and paste recombination between two DNA recognition sequences [28]. The versatile and compact group-II introns of bacteria are known to create genetic diversity from a population of intron invaded mRNAs by circulation and trans-splicing to produce new splicing products with novel functions [29].

In the class of cell signaling we found the presence of ankyrin repeat, methyl-accepting chemotaxis protein (MCP), bacterial SH3 domain and HD-domain (Figure 3 - see PDF). The ankyrin repeat motif is nearly 33 residues long and is the most common protein-protein interaction motif in nature. The interaction of ankyrin repeat with target proteins is mediated by forming helix-turn-helix structure within protein [30]. Ankyrin repeats are functionally diverse and in addition to *O. tsutsugamushi*, also present in other intracellular pathogenic bacteria (Coxiellaburnetii, Rickettsiaspp., and Wolbachiapipientis) [31]. Various intracellular bacterial pathogens translocate ankyrin repeat containing proteins by different secretion systems into host cells, where they manipulate various host processes [30,32]. The chemotaxis MCP protein act as receptor for intracellular and environmental signals and regulates various cellular signaling processes involved in cell survival, pathogenicity and biodegradation [33].

The metabolism class showed the presence of citrate synthase, lipase, glycoside hydrolase family 18, alpha/beta hydrolase fold and uroporphyrinogen III synthase (Figure 3, Table 1 - see PDF). The enzyme of kerbs cycle or central energy metabolism, i.e., citrate synthase is a moonlighting enzyme which act an essential check point transcriptional regulator to control bacterial cell cycle [34]. Lipase is a type of hydrolases which hydrolyzes the ester bonds in monoacylglycerols and have crucial function in fat metabolism [35]. The role of enzyme GH18 is known earlier in hydrolyzing glycosidic bond present between two or more carbohydrates [36].

The transport of category consists of outer membrane enzyme PagP, TolA, MrpF_PhaF, P-loop NTPase and Armdillo-type fold (Figure 3, Table 1 - see PDF). The outer membrane enzyme PagP in gram-negative bacteria is responsible for transferring palmitate chain from phospholipid to glucosamine unit of lipid A, cationic antimicrobial peptides resistance, signal transduction and in evading host immune defense [37,38]. TolA protein interacts with Pal protein to form an inner membrane and outer membrane connection which ultimately involved in antibiotic resistance [39]. MrpF_PhaF is an integral membrane protein which act as a part of potassium efflux pump that involved in regulation of pH [40]. The P-loop NTPase is one of the most abundant monophyletic protein domain present in the proteomes of prokaryotes. The role of P-loop NTPaseshave noticed earlier in numerous processes including transcription, replication, membrane transport, intracellular trafficking and activation of different metabolites [41,42]. Ulp1 protease (ubiquitin-like protein-specific proteases) is the single protein found in the class of proteolysis (Figure 3 - see PDF). Ulp1 is the major proteases which cleave small ubiquitin-related modifiers (SUMO)and play essential role in progression of cell cycle [43, 44].

The metabolic pathway analysis of 44 HPs was carried out at KEGG with the help of BlastKOALA. Two HPs of *O. tsutsugamushi*, i.e., WP_04161591.1 (rpoZ, DNA-directed RNA polymerase subunit omega) and WP_041621613.1 (gltA, citrate synthase) found to be involved in different metabolic pathways. Protein WP_04161591.1 with KEGG orthology number K03060 is involved in RNA polymerase pathway with KEGG pathway ID map03020. The role of rpoZ gene has been noticed earlier in various physiological processes such as formation of biofilm, motality and resistance to antibiotics [45]. The protein WP_041621613.1 with KEGG orthology number K01647 is involved in eight pathways, namely citrate cycle (KEGG pathway ID: map00020), glyoxylate and dicarboxylate metabolism (KEGG pathway ID: map00630), metabolic pathways (KEGG pathway ID: map0100), biosynthesis of secondary metabolites (KEGG pathway ID: map01110), microbial metabolism in diverse environments (KEGG pathway ID: map01120), carbon metabolism (KEGG pathway ID: map01200), 2-oxocarboxylic acid metabolism (KEGG pathway ID: map01210) and biosynthesis of amino acids (KEGG pathway ID: map01230). The gltA is required by pathogenic bacteria to establish the infection in different organs. Earlier, thecitrate synthase is known to conferred metabolic flexibility to pathogen Klebsiella pneumonia which impact site specific fitness during infection [46]. Previous studies reported that the proteins involved in the various metabolic pathways of pathogen may act as probable therapeutic targets and are crucial for pathogens survival [7,47,48]. Therefore, HPs involved in metabolic pathways can serve as the probable drug targets and help in the drug discovery process.

The distribution of functional HPs in the subcellular locations of *O. tsutsugamushi* was predicted by SOSUIGramN. The subcellular localization of 44 HPs showed that 14 were present in cytoplasm, 4 in inner membrane, 8 in outer membrane, 5 in extra cellular space and 13 HPs have unknown location. Figure 4(see PDF) shows the subcellular location of 44 HPs. The precise subcellular localization is an important authentication used for inferring function of the protein. It can also help in designing the lab experiment to study particular protein of interest and may help in the identification of antimicrobial targets, factors contributing to host invasion and potential vaccine candidates [49].

The identification of non-human homologous HPs will enhance the specificity of the designed drug and targeting of host homologous proteins may cause cytotoxicity and cross-reactivity [7]. Hence, we prioritized potential therapeutic targets first by identifying the non-human homologous HPs in *O. tsutsugamushi*. All the 44 functionally annotated HPs were non-human homologous i.e. were present in *O. tsutsugamushi* and absent in host Homo sapiens (Table 2 - see PDF). Essential proteins are imperative for the survival of the organism. The DEG was used to predict the essential proteins in the set of functionally annotated HPs. We have identified 17 HPs which may be essential for the survival of the *O. tsutsugamushi*. Five HPs (WP_012461311.1, WP_012461356.1, WP_012462081.1, WP_012462252.1 and WP_041621613.1) were found essential which may involve in numerous cellular functions and have crucial role in the survival of the pathogen [47].

The physicochemical properties of the HPs were predicted further which are crucial for their structure, activity and biological function. The molecular weight of 44 HPs ranges from 4.68 kDa (WP_148141124.1) to 153.61 kDa (WP_012462337.1). The proteins molecular weight should be ≤ 110 kDa to act better therapeutic targets as earlier studies suggested that small sized proteins are more soluble and can be easily purified in wet lab experiments [50, 51]. Two HPs (WP_012461335.1 and WP_012462337.1) have molecular weight more than 110 kDa. A total of 29 HPs have theoretical pI more than 7 and 15 HPs had theoretical pI less than 7. The predicted pI of HPs can be useful to prepare the buffers for protein purification using isoelectric focusing. The aliphatic index of the HPs ranges from 73 (WP_148141124.1) to 160.12 (WP_041621668.1). The high aliphatic index indicates that these proteins can be stable at wide range of temperature [52,53]. The GRAVY of the HPs were calculated and it ranges from -0.834 (WP_080503986.1) to 1.323 (WP_041621668.1). The low value of GRAVY is the positive indicator for the good interaction of water molecules with proteins.The host cell functions were modulated by bacterial toxins (virulence factor) in order to favor microbial infection [54]. There are different virulence factors which can be cytosolic, secretory and membrane associated. These virulence factors are important constituents of bacteria as they help to undergo morphological and physiological shifts, aid in adherence to host cell or to kill host cells and to resist the host immune response [55][56]. We have predicted that 37 non-human homologous HPs were putative virulent factors that may contribute to pathogenesis of *O. tsutsugamushi* (Table 2 - see PDF). Furthermore, the functional annotation performed in earlier step also strengthen the finding that HPs proteins can act as putative therapeutic targets and play important roles in drug development.

## Conclusion and future perspectives:

*O. tsutsugamushi* is an intracellular pathogen of one of the neglected disease scrub typhus and therefore its genome is less studied by the scientists. More than one quarter of the proteins in O.tsutsugamushi strain Ikeda have unknown function. We report 44 annotated proteins among 241 HPs using systematic Bioinformatics tools. Most of the annotated HPs involved in gene expression and regulation and cell signaling. The categories of metabolism, transport and proteolysis have shared few proteins. These proteins found to be localized in cytoplasm, inner membrane, outer membrane and extra cellular space. Moreover, all the annotated 44 proteins found non-homologous to human and 37 proteins among these 44 proteins have shown virulence activity implying therapeutic nature for further consideration in drug discovery against the pathogen.
